# Effects of stress on behavior and resting-state fMRI in rats and evaluation of Telmisartan therapy in a stress-induced depression model

**DOI:** 10.1186/s12888-018-1880-y

**Published:** 2018-10-17

**Authors:** Junling Li, Ran Yang, Kai Xia, Tian Wang, Binbin Nie, Kuo Gao, Jianxin Chen, Huihui Zhao, Yubo Li, Wei Wang

**Affiliations:** 1 School of Traditional Chinese Medicine, Capital Medical University, Beijing, 100069 China; 2 Beijing University of Chinese Medicine, Beijing, 100029 China; 3 Cardiovascular department of Guang’anmen Hospital, China Academy of Chinese Medical Sciences, Beijing, 100053 China; 4 Key Laboratory of Nuclear Analytical Techniques, Institute of High Energy Physics, Chinese Academy of Sciences, Beijing, 100049 China; 5 Institute of Basic Theory for Chinese Medicine, China Academy of Chinese Medical Sciences, Beijing, 100700 China

**Keywords:** Chronic stress, Telmisartan, fMRI, Resting state, Behavioral test, Reho, ALFF

## Abstract

**Background:**

The etiology of depression and its effective therapeutic treatment have not been clearly identified. Using behavioral phenotyping and resting-state functional magnetic resonance imaging (r-fMRI), we investigated the behavioral impact and cerebral alterations of chronic unpredictable mild stress (CUMS) in the rat. We also evaluated the efficacy of telmisartan therapy in this rodent model of depression.

**Methods:**

Thirty-two rats were divided into 4 groups: a control group(C group), a stress group(S group), a stress + telmisartan(0.5 mg/kg)group (T-0.5 mg/kg group) and a stress + telmisartan(1 mg/kg) group (T-1 mg/kg group). A behavioral battery, including an open field test (OFT), a sucrose preference test (SPT), and an object recognition test (ORT), as well as r-fMRI were conducted after 4 weeks of CUMS and telmisartan therapy. The r-fMRI data were analyzed using the amplitude of low-frequency fluctuations (ALFF) and regional homogeneity (ReHo) approach. The group differences in the behavior and r-fMRI test results as well as the correlations between these 2 approaches were examined.

**Results:**

CUMS reduced the number of rearings and the total moved distance in OFT, the sucrose preference in SPT, and novel object recognition ability in ORT. The telmisartan treatment (1 mg/kg) significantly improved B-A/B + A in the ORT and improved latency scores in the OFT and SPT. The S group exhibited a decreased ReHo in the motor cortex and pons, but increased ReHo in the thalamus, visual cortex, midbrain, cerebellum, hippocampus, hypothalamus, and olfactory cortex compared to the C group. Telmisartan (1 mg/kg)reversed or attenuated the stress-induced changes in the motor cortex, midbrain, thalamus, hippocampus, hypothalamus, visual cortex, and olfactory cortex. A negative correlation was found between OFT rearing and ReHo values in the thalamus. Two positive correlations were found between ORT B-A and the ReHo values in the olfactory cortexand pons.

**Conclusions:**

Telmisartan may be an effective complementary drug for individuals with depression who also exhibit memory impairments. Stress induced widespread regional alterations in the cerebrum in ReHo measures while telmissartan can reverse part of theses alterations. These data lend support for future research on the pathology of depression and provide a new insight into the effects of telmisartan on brain function in depression.

## Background

Depression is a complex psychiatric disorder characterized by anhedonia and feelings of sadness [1]. It is not only life threatening but also has a negative impact on cognitive processes, especially learning and memory [2]. Stress is known to be a key factor in the development of depression and memory impairment. However, the pathogenes of stress leading to depression and its effective therapeutic strategies have not been clearly identified.

There are many limitations in conducting research on the etiology of depression and the efficacy of new drugs on individuals with depression due to ethical reasons. Therefore, it is imperative to use reliable preclinical animal models in order to evaluate effective therapies prior to implementing these strategies in the clinic. One rat model of depression, which was initially described by Willner [3], uses a regimen of chronic unpredictable mild stress (CUMS) to mimic the daily hassles and stress levels in humans. It has been commonly used to study the etiology of depression and antidepressant efficacy [4, 5].

Resting-state functional magnetic resonance imaging (r-fMRI), a promising neuroimaging technique that measures intrinsic or spontaneous neural activity in vivo [6], has been increasingly used to study neuropsychiatric disorders, including mild cognitive impairment (MCI) [7], depression [8], Alzheimer’s disease (AD) [9], schizophrenia [10] and medial temporal lobe epilepsy [11]. In contrast to task-based experimental paradigms, r-fMRI is another way to capture brain activity from rodents that are unable to complete functional tasks due to the anesthesia and/or restraint used during traditional fMRI data acquisition [12]. Analysis of the amplitude of the low-frequency fluctuations (ALFF) and the regional homogeneity (ReHo) are two methods that investigate the resting-state activity in regions across the brain [13, 14]. These two methods have been successfully applied to detect alterations in subjects with various mental disorders [7, 8, 15, 16]. ALFF measures the amplitude of the regional spontaneous neuronal activity [17]. The ReHo method, developed by Zang et al. [14], focuses on the similarities or the coherence of intraregional spontaneous low-frequency activity, measuring the level of coordination in regional neural activity. Hence, ALFF and ReHo, which provide different types of information regarding neuronal activity, are two complementary methods that investigate alterations in the activity of the entire brain. The combination of ALFF and ReHo may provide a more comprehensive pathophysiological assessment of brain dysfunction than either method alone, especially in exploratory research.

Angiotensin type 1 receptor (AT1 receptor) blockers have attracted much attention for their possible antidepressant effects. The renin-angiotensin system (RAS) is one of the critical body reaction systems in response to stress [18]. The pathophysiological response to stressful stimuli that exceeds the body’s adaptive mechanisms include increased brain Angiotensin II (Ang II) activity, amplified AT1 receptor expression, which is associated with higher hypothalamic-pituitary-adrenal (HPA) axis activation, and enhanced peripheral RAS activity [19]. In addition, excessive brain AT1 receptor activity is associated with brain inflammation [20], which is involved in the pathogenesis of emotional and cognitive impairments [21]. Telmisartan, a commonly used angiotensin receptor blockers (ARBs), is very lipophilic, which allows it to readily cross the brain-blood barrier (BBB). Because telmisartan induces central AT1 receptor blockade [22], this compound can be used as a potential oral antidepressant. Several studies have demonstrated that telmisartan is neuroprotective [23] and can attenuate cognitive impairments induced by chronic stress in rats [24]. However, there are few reports to explore which brain regions telmisartan’s possible antidepressant effect is related to.

Based on the previous reports, we hypothesized that telmisartan could alleviate the depressive and cognitive dysfunction symptoms caused by chronic stress. The related brain regions could be explored by the ALFF and Reho analysis for r-fMRI. Hence, we employed the two analyses to directly compare the resting-state brain activity among normal rats, rats that were exposed to chronic stress, and rats exposed to chronic stress and administered telmisartan. In addition, the open field test (OFT), the sucrose preference test (SPT), and the object recognition test (ORT) were conducted to evaluate locomotor activity, anhedonia, and cognition in the experimental animals.

## Methods

### Animals

These experiments were conducted in male Sprague-Dawley rats (5 weeks), provided by Beijing Weitong Lihua Experimental Animal Technology Co., Ltd. (experimental animal production license: SCXK Beijing 2012–0001). The rats were housed 4 per cage in a temperature-(18–24 °C) and humidity-(40–60%) controlled room on a 12 h light/dark cycle (lights on at 7:00 a.m.). The rats had free access to standard laboratory food and tap water. Animal maintenance was performed according to the National Institutes of Health Guidelines for the Care and Use of Laboratory animals [25].The experimental protocols were approved by the Beijing University of Chinese Medicine Institutional Animal Care and Use Committee (Ethics number: 2013BZHYLL1001B). All efforts were made to minimize animal suffering. All animals were decapitated after anesthetized deeply with isoflurane.

### Drug administration and experimental groups

After 2 weeks of acclimation, 32 rats were randomly divided into 4 groups with 8 animals per group including: (1) a Control group (C group)that was administered distilled water as a vehicle; (2) a Stress group (S group)that underwent the CUMS procedure and was administered distilled water; (3) a Stress + Telmisartan group (T-0.5 mg/kg group) that underwent the CUMS procedure and was administered telmisartan (0.5 mg/kg) dissolved in distilled water; (4) a Stress + Telmisartan group (T-1 mg/kg group) that underwent the CUMS procedure and was administered telmisartan (1 mg/kg) dissolved in distilled water. The telmisartan dose of 1 mg/kg is considered to be a nonhypotensive dose in rats [26, 27]. The rats received either telmisartan or vehicle by oral gavage each day immediately before the stress procedure.

### Chronic unpredictable mild stress (CUMS) procedures

The rats in the C group were housed in groups of 4, while the rats in the S and T groups were singly housed in isolation. The following stimulations were applied to the experimental animals, according to previous CUMS rat model methods. These included 12 h food deprivation, 12 h water deprivation, overnight wet housing, forced swimming (4 °C for 5 min), 2.5 h restraint, overnight illumination, 45°cage tilt for 12 h, and 36 sessions of inescapable foot shock (1.5 mA intensity; 30 shocks in 1 min, with an inter-session interval of 30s). The rats were exposed to a random selection of 2 stressors per day, with no repetition of the same type on continuous days, in order to have the stimulation remain unpredictable. The CUMS process lasted for 4 continuous weeks. The behavioral experimental tests were performed 24 h after the last stimulation. There were no rats injured or ill during the experiment.

### Open field test (OFT)

The open field test was performed to assess the rats’ spontaneous exploratory activity. The open field arena was a square 100 cm × 100 cm black floor divided by 8 lines into 25 equal squares, surrounded by a 35 cm high wall. A digital camera was placed 2 m above the open field to capture the whole field. During OFT, the rats were placed in the center of the field and recorded using a small animal behavior recorder for 3 min. The rearings were counted manually during the recording. An animal behavior analysis system was used to analyze the total moved distance during the 3 min.

### Sucrose preference test (SPT)

The sucrose preference test evaluated potential anhedonia in the experimental animals. The rats were trained for the SPT by providing a continuous choice of 2 bottles, which contain 2% sucrose, for 24 h. Afterwards, one of the bottles was replaced with water for 24 h. During this 24-h period, the bottles were switched after 12 h to control for any side bias. Following this adaptation procedure, the rats were deprived of water and food for 12 h. The SPT was conducted at 9:00 a.m. The rats were housed in individual cages and given free access to the 2 bottles of water and sucrose, which were weighed in advance. After 4 h, the weight of both the consumed sucrose solution and water was recorded. The sucrose preference was calculated as sucrose preference (%) = sucrose consumption (g)/(sucrose consumption (g) + water consumption (g)) × 100%.

### Object recognition test (ORT)

Object recognition was evaluated in a plastic box 62 cm long, 40 cm wide and 45 cm high. The objects, which were in duplicate, were made of glass, and did not appear to have any innate significance for the subject animals. The rats were naïve to the objects, which were weighted so they could not be displaced by the test subject. ORT was performed as described previously [28]. Briefly, all rats underwent 2 habituation sessions with a 1 h inter-session interval. During habituation, the animals were allowed to freely explore the apparatus for 3 min. After 24 h, the rats returned to the testing apparatus for the experimental session. The experimental session consisted of 2 trials, which were 3 min and 5 min in duration, respectively. During the first trial (T1), the rats were exposed to 2 identical objects, A1 and A2. During the second trial (T2) 60 min later, the rats were exposed to 2 objects, a duplicate of either A1 or A2, and a new object, B. The position of the 2 objects was counter balanced and randomly permuted during T2 in order to reduce any bias due to object and place preference. Object recognition was assessed by the subject rat’s T2 object exploration time. The object exploration was defined as the subject touching the object with its nose. Turning around or sitting on the object was not considered to be exploratory behavior. The time spent in exploring the 2 different objects in T2 was recorded. Object recognition was defined as variable B-A, and B-A/B + A.

### fMRI acquisition

fMRI measurements were conducted on a 7.0 T /16US MRI scanner (Bruker), using a radiofrequency transmission coil (300 1H 089/072 QUAD TO AD) and a 38 mm rat head surface coil for receiving. The rats were anesthetized with isoflurane/O2 (5% for induction and 1–1.5% for maintenance) and prostrated on a custom-made holder to minimize any head motion. The respiration was monitored at a rate of 40–50 breaths per min. First, the T2-weighted data were acquired for the localization of the functional scans. Then, the resting-state functional images were recorded axially over 13 min and 26 s with the following parameters: repetition time (TR) = 3280 ms, Matrix = 128*128, echo time (TE) = 27.6 ms, flip angle = 90°, 20slices ,thickness/gap = 1.0 mm/0.2 mm, field of vision (FOV) =2.5 cm × 2.0 cm.

## Statistical analysis

### Behavior test data analysis

SPSS 17.0 software (SPSS v.17.0 for Windows; SPSS Inc., Chicago, IL, USA) was used to analyze the data. A Shapiro-Wilk test was used to examine the normality of the data. If the data fit a normal distribution, a one-way analysis of variance (ANOVA) was used to compare the 4 groups. For a comparison of 2 selected groups, an LSD post-hoc test was used for data with a homogeneity of variance, and a Tamhane’s T2 test was used for those with a heterogeneity of variance. If the data did not have a normal distribution, a Kruskal-Wallis H test was used. A *p* ≤ 0.01 was considered significant.

### fMRI data analysis

The data were pre-processed using spmratIHEP based on the statistical parametric mapping (SPM8) software and Resting-State fMRI Data Analysis Toolkit (REST) software, and statistically analyzed by spmratIHEP based on SPM8. The ReHo and ALFF measures were analyzed and compared between the C, S, and T groups.

All the functional images post-processing was performed by a single experienced observer, unaware to whom the scans belonged. The pre-processing and data analysis were performed using spmratIHEP [29, 30], based on SPM8 (Welcome Department of Imaging Science; http://www.fil.ion.ucl.ac.uk/spm) and REST software (http://restfmri.net/forum/index.php?q=rest).

The voxel size of the functional datasets of all individuals were first multiplied by a factor of 5 to better approximate human dimensions, and then pre-processed using the following main steps. (1) Slice timing: the differences of slice acquisition times of each individual were corrected using slice timing. (2) Realign: the temporal processed volumes of each subject were re-aligned to the first volume to remove any head motion, and a mean image was created over the 180 re-aligned volumes. All participants had less than 1 mm of translation in the x, y, or z axis and a 1° of rotation in each axis. (3) Spatial normalization: the re-aligned volumes were spatially standardized into the Paxinos & Watson space [31] by normalizing with the EPI template via their corresponding mean image. Subsequently, all the normalized images were re-sliced by 1.0 × 1.5 × 1.0 mm^3^ voxels (after zooming). (4) Smooth: the normalized functional series were smoothed with a Gaussian kernel of 2mm^3^ Full Width at Half-maximum (FWHM). (5) Removal of the linear trend: the smoothed images had any systematic drift or trend removed using a linear model. (6) ALFF: the filtered estimated the value of LFF. (7) ReHo: It was calculated based on the step (3). In detail, the normalized images had any systematic drift or trend removed using a linear model. Then, the temporal band-pass filtering was performed in order to reduce the effects of low-frequency drift and high-frequency. Finally, the Kendall’s coefficient was calculated to examine the degree of regional synchronization, including the 27 pixels of the fMRI time courses.

The pre-processed images were analyzed within spmratIHEP in SPM8 based on the framework of the general linear model. In order to identify the differences of the ALFF and ReHo measures between the C,S and T groups, a one-way ANOVA was performed. The cerebral regions with significant ALFF and ReHo values between 2 chosen groups were yielded based on a voxel-level height threshold of *p* < 0.001 (uncorrected) and a cluster-extent threshold of 20 voxels.

### Correlations between behavior and fMRI

In order to examine the associations between the evaluated behaviors and the cerebral alterations caused by stress and telmisartan administration, we performed Pearson (data fit a normal distribution) or Spearman (data did not have a normal distribution) correlation analyses between the behavioral test indices and ALLF/ReHo values in the brain regions that exhibited significant differences between the S and C groups, as well as between the S and T groups. A *p* ≤ 0.05 was considered significant. The ALLF and ReHo values were comprised of the mean value of the selected cerebral regions extracted from the smoothed individual images, which were also in the Paxinos & Watson space.

## Results

### The effects of stress and telmisartan on the OFT

**Fig. 1:**
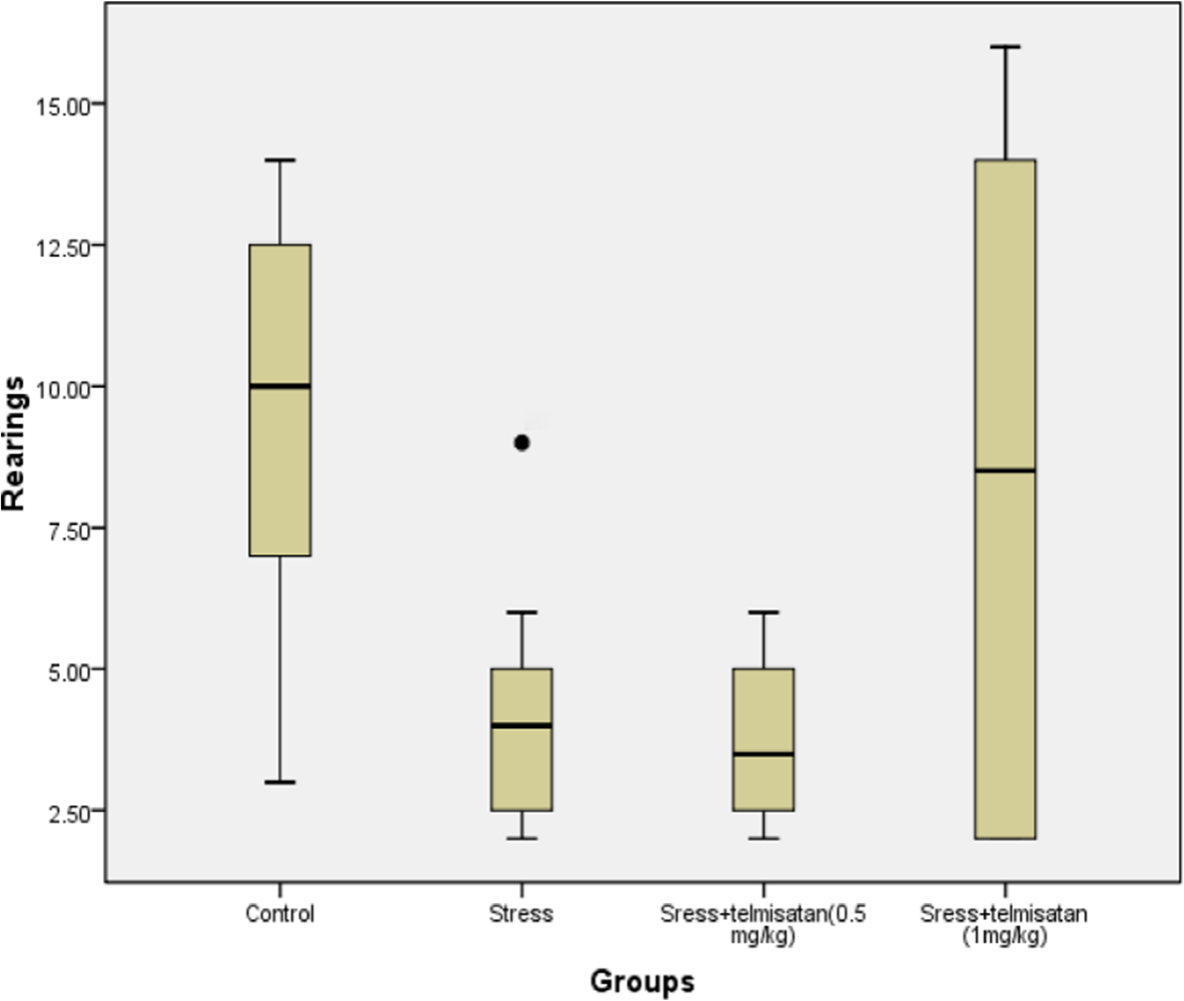
Effects of stress and telmisartan on rearings (outliers)

**Fig. 2:**
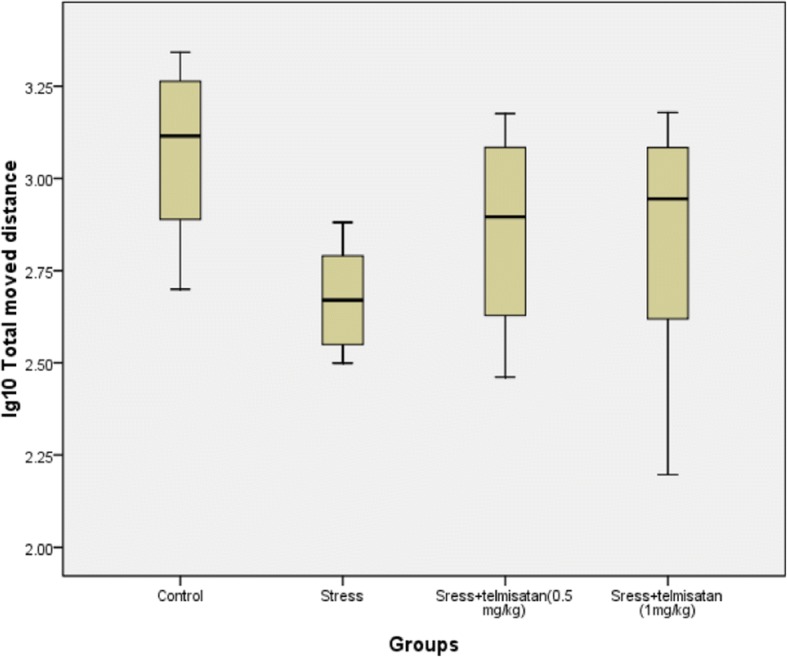
Effects of stress and telmisartan on the total moved distance (unit: centimeter)

The number of rearings in all groups fit a normal distribution with heterogeneity of variance. The Tamhane’s T2 test was used to compare the 2 chosen groups. The data of total moved distance was converted to log10 format to make each group fit a normal distribution with homogeneity of variance. The LSD post-hoc test was used to compare the 2 chosen groups. The total distance was significantly decreased in the S group compared to the C group, while the number of rearings showing a decreased trend (Figs. 1 and 2, Table 1). However, there were no significant differences between theT-1 mg/kg and C groups (Figs. 1 and 2, Table 1). The T-1 mg/kg group exhibited a much higher number of rearings and moved a greater distance than the S group, although these measures were not significant between the two groups (Figs. 1 and 2, Table 1). The T-0.5 mg/kg group only exhibited a much greater moved distance than the S group with no significant difference (Fig. 2, Table 1).

**Table 1:** Effects of stress and telmisartan on the OFT

Rearings	*F* value		*P* value	
	5.976		0.00	
		Effect size	Standard error of effect size	*P* value
	Control-Stress	5.25	1.54	0.03
	Control-Sress+telmisatan (0.5 mg/kg))	5.75	1.42	0.02
	Control-Sress+telmisatan (1 mg/kg))	1.13	2.51	1.00
	Stress-Sress+telmisatan (0.5 mg/kg))	0.50	1.00	1.00
	Stress-Sress+telmisatan (1 mg/kg))	−4.13	2.29	0.49
	Sress+telmisatan (0.5 mg/kg)-Sress+telmisatan (1 mg/kg)	−4.63	2.21	0.35
Lg10 (Total moved distance)	*F* value		*P* value	
	3.28		0.04	
		Effect size	Standard error of effect size	*P* value
	Control-Stress	0.40*	0.13	0.00
	Control-Sress+telmisatan (0.5 mg/kg))	0.22	0.13	0.10
	Control-Sress+telmisatan (1 mg/kg))	0.24	0.13	0.07
	Stress-Sress+telmisatan (0.5 mg/kg))	−0.18	0.13	0.16
	Stress-Sress+telmisatan (1 mg/kg))	−0.16	0.13	0.22
	Sress+telmisatan (0.5 mg/kg)-Sress+telmisatan (1 mg/kg)	0.02	0.13	0.86

Note: *P ≤ 0.01

### The effects of stress and telmisartan on the SPT

**Fig. 3:**
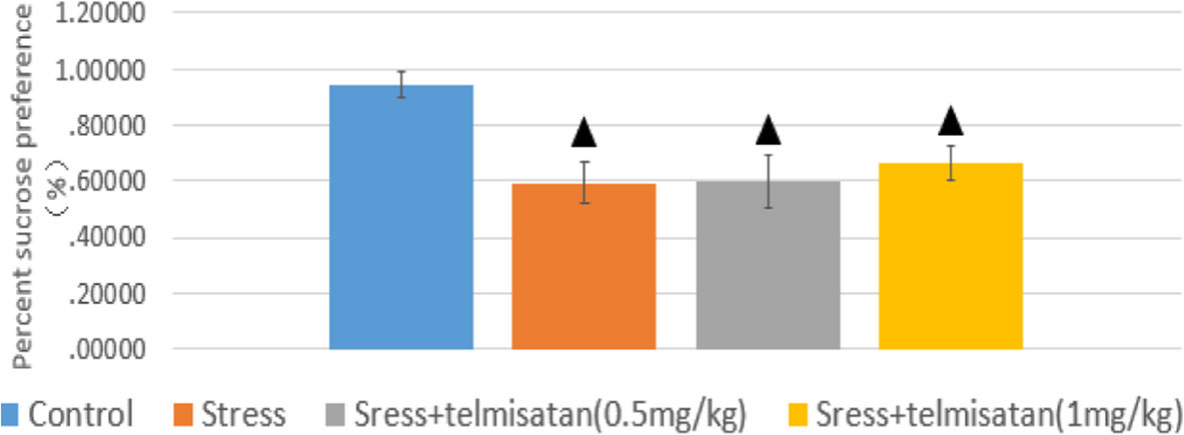
Effects of stress and telmisartan on the SPT. ▲ *p* ≤ 0.01 in comparison to C group, values are means±standard deviation (unit: %)

The sucrose preference measures from all groups fit a normal distribution pattern with a homogeneity of variance. The LSD was then used to compare the 2 chosen groups, followed by the ANOVA. The sucrose preferences were significantly lower in the S, T-0.5 mg/kg, and T-1 mg/kg groups than in the C group (Fig. 3). The telmisartan administration tended to improve sucrose preference, but this was not significant when compared to the S group.

### The effects of stress and telmisartan on the ORT

**Fig. 4:**
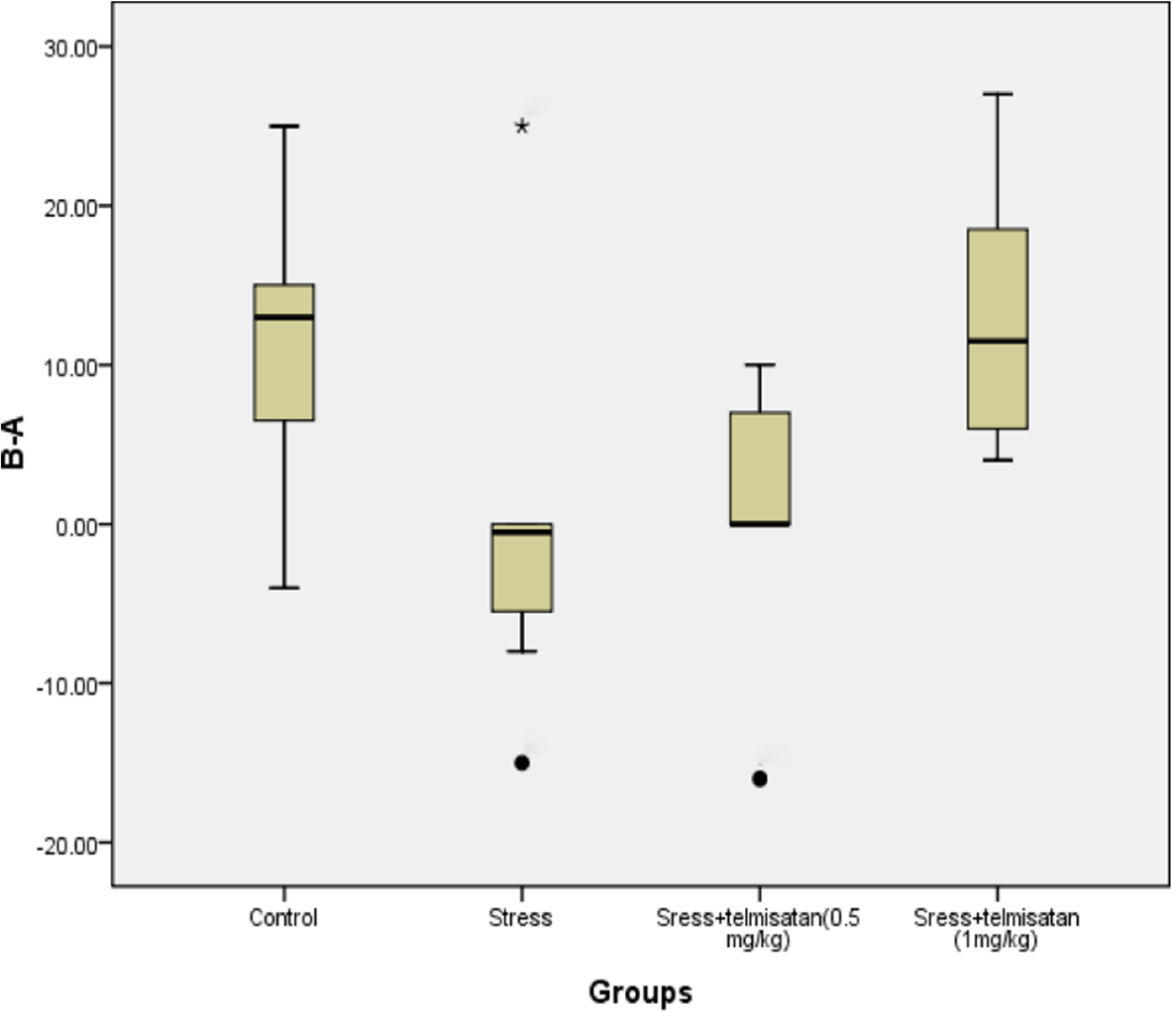
Effects of stress and telmisartan on the B-A (outliers, *extreme values)

**Fig. 5:**
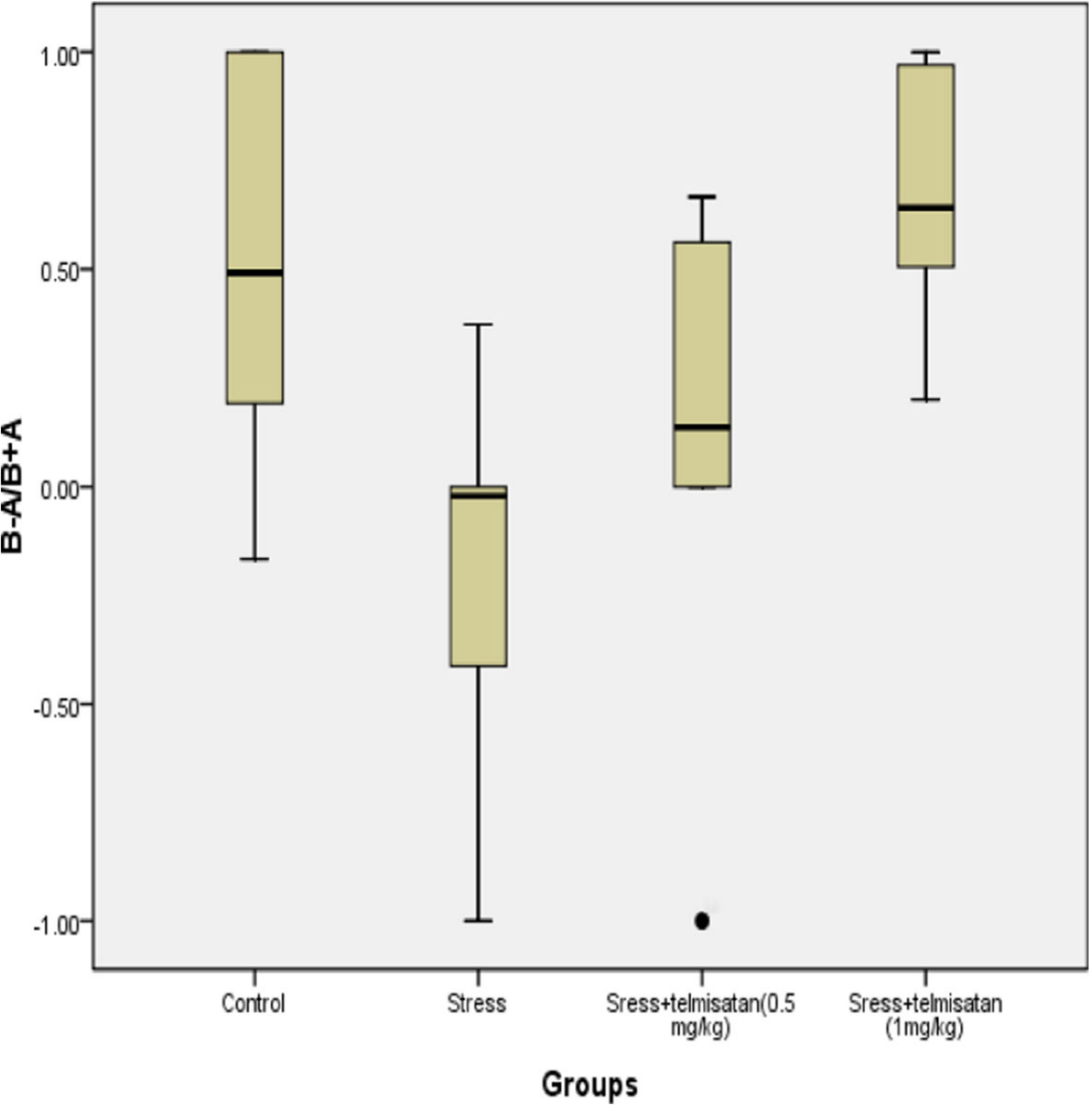
Effects of stress and telmisartan on the B-A/B + A (outliers)

A Shapiro-Wilk test indicated that the B-A data in the S group was not normally distributed. A Kruskal-Wallis H test compared the B-A values among the 4 groups. The result was that the x^2^ = 11.96,*p* = 0.00. The mean rank in each group (from high to low) was as follows: T-1 mg/kg > C > T-0.5 mg/kg > S (Fig. 4, Table 2). To some extent, these results suggest that stress caused an object recognition memory impairment and that telmisartan can attenuate this impairment. The data of B-A/A + B in each group was normally distributed, and had a homogeneity of variance. The B-A/A + B in the S group was significantly lower than the C group (Fig. 5, Table 2). The B-A/A + B of the T-1 mg/kg group was not significantly different from the C group (Table 2), but was significantly higher than that of the S group (Table 2), suggesting that telmisartan at the dose of 1 mg/kg reversed the stress-induced object recognition memory impairment. The T-0.5 mg/kg group exhibited a higher B-A/B + A than S group but with no significant difference.

**Table 2:** Effects of stress and telmisartan on the ORT

B-A/B + A	*F* value		*P* value	
	6.5		0.02	
		Effect size	Standard error of effect size	*P* value
	Control-Stress	0.71*	0.22	0.00
	Control-Sress+telmisatan (0.5 mg/kg))	0.39	0.22	0.08
	Control-Sress+telmisatan (1 mg/kg))	−0.15	0.22	0.48
	Stress-Sress+telmisatan (0.5 mg/kg))	−0.32	0.22	0.15
	Stress-Sress+telmisatan (1 mg/kg))	−0.87*	0.22	0.00
	Sress+telmisatan (0.5 mg/kg)-Sress+telmisatan (1 mg/kg)	−0.55	0.22	0.02
B-A	x2 value		*P* value	
	11.99		0.01	
			Mean Rank	
	Control		21.25	
	Stress		9.56	
	telmisatan (0.5 mg/kg)		12.25	
	telmisatan (1 mg/kg		22.94	

Note: *P ≤ 0.01

### fMRI

According to the result of behavior tests, the telmisartan administration at the dose of 1 mg/kg significantly improved the behavior changes caused by stress, while the effect of telmisartan administration at the dose of 0.5 mg/kg was weak. Hence, the r- fMRI was conducted on the rats in the C, S and T-1 mg/kg groups to investigate the effects of stress and telmisatan administration on the brain.

#### ALFF

**Fig. 6:**
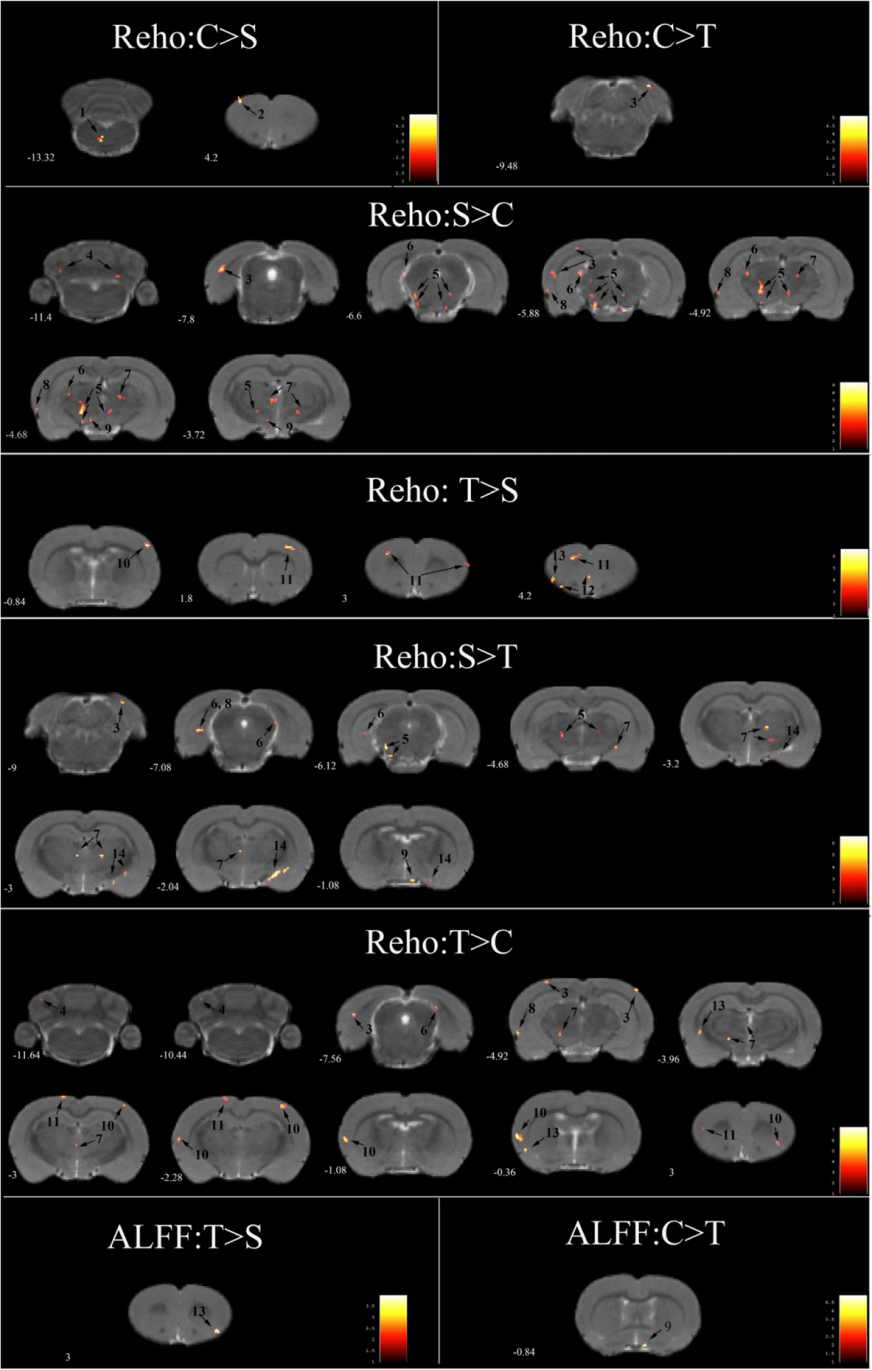
Statistical maps of voxel t values of ReHo and ALFF comparisons of two chosen groups. The numbers at the bottom left of each image refer to the z coordinates in the stereotaxic space of Paxinos and Watson. The color bars were used to signify the t-value of the group analysis(the color is brighter, the t value is higher). The left side of the images corresponds to the left side of the brain, and vice versa. The numbers on each brain image stand for the different brain regions as follows: 1. pons 2. motor cortex 3.visual cortex 4. cerebellum 5. midbrain 6. hippocampus 7. dorsal thalamus 8. olfactory cortex 9. hypothalamus 10. sensory cortex 11.motor cortex 12.orbital cortex 13. insular cortex 14. amygdaloid body

There were no significant alterations in ALFF between the C and S groups. The T group exhibited an increased ALFF in the insular cortex compared with the S group, and a decreased ALFF in the hypothalamus compared with the C group (Fig. 6).

#### ReHo

There were widespread cerebral region alterations in the ReHo measures between the C, S, and T-1 mg/kg groups (Fig. 6). The S group demonstrated a decreased ReHo in the motor cortex and pons, an increased ReHo in the thalamus, visual cortex, midbrain, cerebellum, hippocampus, hypothalamus, and olfactory cortex compared to the C group. Conversely,the motor cortex in the T-1 mg/kg group displayed an increased ReHo compared with the S and C groups, suggesting that telmisartan reversed the stress-induced alterations in the motor cortex. The midbrain, thalamus, hippocampus, hypothalamus, visual cortex, and olfactory cortex in the T-1 mg/kg group exhibited a decreased ReHo compared to the S group, and an increased ReHo or no change compared to the C group, suggesting that telmisartan attenuated or eliminated the corresponding cerebral alterations caused by stress. The other cerebral regions, including the insular cortex, sensory cortex, orbital cortex, and amygdaloid body, were significantly different in their ReHo measures between the T-1 mg/kg and S groups but were not different between the C and S groups.

### Correlations between behavior and fMRI

The regions with significant group differences (S vs. C) in ReHo included the motor cortex, pons, thalamus, visual cortex, midbrain, cerebellum, hippocampus, hypothalamus, and olfactory cortex. The brain regions showing significant difference between the T-1 mg/kg and S groups in ALFF or ReHo included the insular cortex, sensory cortex, orbital cortex and amygdaloid body. We analyzed the correlations between the OFT and ORT’s indices and the ALLF and Reho values in the altered brain regions. The significant correlations were between the number of rearings in the OFT and the ReHo values in the thalamus (*r* = − 0.446, *p* = 0.029), the value of B-A and the ReHo values in the olfactory cortex(*r* = 0.501, *p* = 0.013) and pons(*r* = .413, *p* = 0.045).

## Discussion

The present study investigated the behavioral and cerebral alterations induced by chronic stress and telmisartan administration. To the best of our knowledge, this is the first study using ALFF and ReHo methods in a rat model of depression to examine whole brain r-fMRI alterations caused by stress and telmisartan administration.

According to our behavioral results, chronic stress decreased locomotor activity, sucrose preference and impaired ability of novel object recognition, which is in line with the results of previous studies, and suggests that we successfully established a rat model of depression [24, 32]. Since the dose of 1 mg/kg/day telmisartan has been proven nonhypotensive for mice [26], we applied two doses’ (1 mg/kg/day and a lower dose, 0.5 mg/kg/day) telmisartan on the model. We found that the telmisartan of 1 mg/kg /day could significantly improve the impaired ability of novel object recognition, while the effect of 0.5 mg/kg/day was weak. Since the ORT indicating a kind of cognitive function, it’s worth conducting additional experiments such as Morris water maze to evaluate telmisartan’s potential effect on the cognitive function more comprehensively. For the tests of OFT and SPT, we found a trend for telmisartan to improve the decreased locomotor activity and sucrose preference, although these were not significant. A previous study showed that the treatment with telmisartan of 1 mg/kg for five weeks could notably improve the depressive symptoms in the stress mouse model [33]. Hence, the not significant effect may be due to the short treatment time and a relatively small dose for rats. Taken together, these data demonstrated a possible antidepressant effect with telmisartan. It’s worth performing further analyses to look into the potential of telmisartan in alleviation of symptoms of depression.

We observed no significant alterations in ALFF in the chronically stressed rat compared to the normal rat. However, there were widespread cerebral region alterations in ReHo between the two groups. To some extent, it can be concluded that the coordination of regional neural activity, rather than its amplitude, may be more easily disrupted by chronic stress.

In the present study, we observed that the rats in the S group had increased ReHo in some limbic system regions, including the hippocampus, hypothalamus,thalamus, and olfactory cortex. The hippocampus, a critical structure in the limbic system, is well-known for its important role in the formation of depression [34] and cognitive decline [35]. The result of increased ReHo value in the hippocampus is consistent with a previous study on the later adult onset depression patients, supporting the validity of our findings [36]. There were rare clinical reports about the altered ReHo in the hypothalamus, though hypothalamus’ pathological alterations have been widely reported in depression and cognitive impairment [37–39]. Hence, there may be differences between the rat model and patients with depression in the Reho signal of hypothalamus. Thalamus has been well positioned for an involvement in major depressive disorder pathophysiology, earning its name as the limbic thalamus [40]. Previous study has showed that cognitive decline may be related to the volume of thalamic atrophy [41]. In the present study, we found increased Reho within the thalamus in the S group, and notably, a significant negative correlation between the number of rearings in the OFT and the ReHo within the thalamus. Since OFT rearings negatively correlated with the emotionality and anxiety level in rats [42], our study demonstrated that increased Reho within the thalamus could be served as a potential neuroimaging marker for the depression**.** It has been found that the dysfunction of olfactory cortex was closely related with the cognitive impairment [43]. This study also found a significant positive correlation between the value of B-A and the ReHo values in the olfactory cortex, further confirmed the important role of olfactory cortex in the cognitive function.

In addition to a direct involvement of limbic system, our results demonstrated stress-induced ReHo alterations in other cerebral regions, including the cerebellum, pons, motor cortex, visual cortex, and midbrain. Previous studies have demonstrated ReHo alterations in the pons and cerebellum in depressed patients [15, 44–46], which is consistent with our study. Together, these data suggest that the pons and cerebellum are associated with depression. These abnormalities could serve as markers for the diagnosis of depression and are worth exploring further for their role in the etiology of depression. Besides, since a significant positive correlation was found between the B-A value and the ReHo within the pons, the decreased ReHo in pons could also be a marker of cognitive impairment. The function of the motor cortex has been shown altered in depression [47], and a decreased ReHo within motor cortex was found in the generalized anxiety disorder patients [48]. Hence, the result of decreased ReHo within motor cortex in the present study may be a neuroimaging reflection of anxiety. Although several studies have demonstrated abnormal cerebral connectivity involving the visual cortex and midbrain in depression [49, 50], rare clinical studies reported altered ReHo in the two regions. Since the pathological alterations of the visual cortex and midbrain exist in depression [51–53], there may be also differences between the rat model and patients with depression in the visual cortex and midbrain Reho signal.

Telmisartan has strong anti-inflammatory effects on the brain [23, 54] and neuroprotective effects in cultured primary neurons [55], suggesting that telmisartan may be a novel therapeutic approach for the treatment of depression. Some studies suggest that telmisartan can ameliorate memory deficits through beneficially effects on the hippocampus [56, 57], little is known about which other brain regions are affected by telmisartan. Using ALFF and ReHo, our study demonstrated that stress-induced alterations in multiple brain regions were partly reversed or attenuated by telmisartan. In addition to decreased ReHo measures in the motor cortex and pons due to stress, the motor cortex in the T-1 mg/kg group displayed an increased ReHo compared to the S and C groups, suggesting that telmisartan reversed the motor cortex changes caused by stress. Increased ReHo in the midbrain, thalamus, hippocampus, hypothalamus, visual cortex, and olfactory cortex caused by stress was identified. However, the T-1 mg/kg group exhibited a decreased ReHo in these regions compared to the S group, and increased or unchanged ReHo in these regions compared to the C group. Hence, telmisartan also attenuated or eliminated these stress-induced brain region changes. Besides, since a significant negative correlation was found between the number of rearings in the OFT and the ReHo within the thalamus, telmisartan may attenuate the anxiety symptoms by decreasing the hypercoordination of neural activity within the thalamus. Thalamus could be an important brain region to investigate further in the future study. Other brain regions, including the insular cortex, the sensory cortex, the orbital cortex, and the amygdaloid body, also showed significant differences between the T-1 mg/kg and S groups. However, because there were no significant differences between the S and C groups, it is difficult to determine whether telmisartan had positive or negative effect on these brain regions. Together, our study demonstrated that telmisartan may be a potential antidepressant by beneficially affected a series of brain regions. Future research is required to determine the extent of telmisartan’s effects on the brain in depression.

In conclusion, our study demonstrated widespread alterations in brain regions in a rat model of depression using the ReHo method. These regions consist of some components of the limbic system that contribute to the formation of depression, but also some other regions, such as the motor cortex, visual cortex, midbrain, cerebellum and pons, which are rarely intensively studied for their role in depression. Combined with previous clinical studies, this study revealed that the Reho alterations in several brain rigions could be potential markers for depression and cognitive impairment. Hence, this study suggests future areas for the pathological research of depression. This study also demonstrated that telmisartan can be a complementary medicine for patients with depression, and affects several brain regions. To our best knowledge, this is the first study of the telmisartan’s antidepressant effect on the whole brain in a rat model of depression.

There were several limitations in our study. Firstly, the rats we used are outbred. Since genetic differences may contribute to the behavioral effects of rodents in models of depression [58], it would be more ideal to use inbred strains in the future. Secondly, we were unable to have a pre- and post-stress brain comparison due to laboratory regulations limiting the physical movement of the animals after r-fMRI. Thirdly, we only observed telmisartan’s effect on the stress model due to our strict animal ethic regulations of limiting the animal number. It will be more rigorous to conduct additional groups to explore whether telmisartan could alter behavior, cognitive function or brain activity in the absence of stress for further study.

## Conclusions

Despite these limitations, this study has demonstrated a series of brain regions involved in depression and telmisartan administration. We believe that this finding has instructive significance for future pathological research of depression and can provide important insight into the effects of telmisartan on nervous system function in depression.

## Abbreviations

AD: Alzheimer’s disease
ALFF: amplitude of low-frequency fluctuations
AngII: Angiotensin II
ANOVA: Analysis of variance
ARBs: Angiotensin receptor blockers
AT1 receptor: Angiotensin type 1 receptor
BBB: Brain-blood barrier
C group: Control group
CUMS: Chronic unpredictable mild stress
FOV: Field of vision
FWHM: Full Width at Half-maximum
HPA: Hypothalamic-pituitary-adrenal
MCI: Mild cognitive impairment
OFT: Open field test
ORT: Object recognition test
RAS: Renin-angiotensin system
ReHo: Regional homogeneity
r-fMRI: Resting-state functional magnetic resonance imaging
S group: Stress group
SNc: Substantia nigra pars compacta
SPT: Sucrose preference test
T-0.5 mg/kg group: Telmisartan(0.5 mg/kg)group
T-1 mg/kg group: Telmisartan(1 mg/kg)group
T1: The first trial
T2: The second trial
TE: Echo time
TR: Repetition time
VAT: Ventral tegmental area
